# Exploratory Clinical and Cost Evaluation of Robot-Assisted Adrenalectomy Using the hinotori™ Surgical Robotic System: A Single-Center Retrospective Study

**DOI:** 10.7759/cureus.113326

**Published:** 2026-07-24

**Authors:** Kotaro Obayashi, Takuya Nishino, Mami Takadate, Hiroya Hasegawa, Hikaru Mikami, Hayato Takeda, Yuki Endo, Yuka Toyama, Takayuki Takahashi, Jun Akatsuka

**Affiliations:** 1 Urology, Nippon Medical School, Tokyo, JPN; 2 Health Policy and Management, Nippon Medical School, Tokyo, JPN; 3 Urology, Nippon Medical School Hospital, Tokyo, JPN; 4 Biomedical Spatial Science, RIKEN Center for Advanced Intelligence Project, Tokyo, JPN

**Keywords:** adrenalectomy, costs, hinotori, minimally invasive surgery, robotic surgery

## Abstract

Purpose: Although the hinotori™ surgical robotic system is Japan's first domestically developed platform, evidence regarding its clinical feasibility remains limited. Because adrenalectomy is a standardized minimally invasive procedure with well-defined perioperative outcomes, it provides an appropriate model for evaluating newly introduced robotic platforms. We evaluated the clinical outcomes and medical costs of robot-assisted adrenalectomy (RAAD) using the hinotori™ system (H-RAAD) and compared them retrospectively with those of da Vinci Xi™-assisted RAAD (D-RAAD). Materials and methods: We retrospectively examined patients who underwent RAAD at Nippon Medical School Hospital between September 2023 and May 2025. Baseline demographic characteristics, perioperative variables, postoperative outcomes, pathological findings, and medical costs extracted from the Diagnosis Procedure Combination (DPC) database were evaluated. Results: We evaluated 12 patients (H-RAAD, n = 5; D-RAAD, n = 7). Baseline characteristics were comparable between the groups, although the median tumor diameter was numerically smaller in the H-RAAD group. The median anesthesia, operative, and console times in the H-RAAD group were 264 (253-307, U = 10.0, p = 0.22), 189 (156-264, U = 10.0, p = 0.29), and 108 (71-180, U = 14.0, p = 0.57) minutes, respectively, comparable with those in the D-RAAD group. Total medical costs per case were similar between the D‐RAAD ($6,333.1) and H‐RAAD ($6,398.4) groups (U = 23.0, p = 0.34). Conclusions: In this small cohort, H-RAAD was completed safely without conversion or major complications, demonstrating perioperative outcomes similar to those of D-RAAD. Although individual cost components varied, total reimbursed medical costs under the DPC system were comparable. Given the limited sample size and exclusion of robotic system capital and maintenance costs, these findings remain exploratory.

## Introduction

With advances in imaging, adrenal tumors, including incidentalomas, functional lesions, hyperplasias, and carcinomas, are increasingly detected. Adrenalectomy remains the standard treatment when hormonal activity or malignancy is suspected. Introduced in 1992, laparoscopic adrenalectomy is well established as a minimally invasive standard [1,2], whereas robot-assisted adrenalectomy (RAAD) has recently emerged as a promising alternative [3,4].

Robot-assisted surgical (RAS) systems provide surgeons with high-definition, three-dimensional visualization, enhanced ergonomics, and improved dexterity through wristed instruments and stable camera control. RAAD is gaining prominence, expanding the operative techniques available to endocrine surgeons and urologists while addressing several inherent limitations of conventional laparoscopic procedures. RAAD yields perioperative outcomes comparable to those of conventional laparoscopic adrenalectomy [5-8]. Recent evidence, including systematic reviews and meta-analyses, suggests that RAAD may offer perioperative advantages over laparoscopic and open adrenalectomy, although findings remain heterogeneous across studies [9-12]. However, these outcomes mostly derive from analyses of procedures performed using the da Vinci Xi™ system (Intuitive Surgical Operations, Inc., Sunnyvale, CA, USA).

Since gaining regulatory clearance from the United States Food and Drug Administration at the turn of the century, the da Vinci™ system has dominated the field of RAS, establishing itself as the cornerstone for most published clinical data. Recently, however, the global market has seen a surge in alternative surgical robotic platforms boasting distinct technological advancements [13-20]. Notable examples that have entered clinical practice encompass the Hugo™ (Medtronic plc, Minneapolis, MN, USA) [13], Senhance™ (Asensus Surgical US, Inc., Durham, NC, USA) [14], KangDuo™ (KangDuo Robot Co., Ltd., China) [15], Revo-i™ (Meere Company Inc., Seoul, Republic of Korea) [16], and Mantra™ (SS Innovations International Inc., Gurugram, India) systems [17]. The hinotori™ (Medicaroid Corporation, Kobe, Japan) was developed in 2020 as Japan's first domestically produced surgical robotic system (SRS). Unlike the da Vinci Xi™ system, the hinotori™ platform features independently movable robotic arms, adjustable arm bases, and a flexible docking configuration designed to reduce arm collision and improve positioning. The modular arm design and flexible docking configuration of the hinotori™ system may contribute to its operative usability and versatility, although further technical evaluation is warranted. Because only a few reports on RAAD using the hinotori™ system (H-RAAD) have been published [18-20], clinical evidence for this platform remains insufficient. Furthermore, the substantial cost of SRS continues to be a major concern for policymakers, healthcare providers, and patients.

This study aimed to evaluate the perioperative outcomes and reimbursed medical costs of H-RAAD compared with da Vinci Xi™-assisted RAAD (D-RAAD).

## Materials and methods

Study design and patient population

This retrospective clinical study was conducted at the Nippon Medical School Hospital (NMSH) in Tokyo, Japan. The study population comprised patients who underwent RAAD for adrenal tumors between September 2023 and May 2025. Twenty consecutive patients were enrolled in the study; however, eight were excluded: five due to incomplete data regarding non-insured costs and three due to planned intensive care unit (ICU) admission for pheochromocytoma or related conditions, because ICU admission substantially influences hospitalization costs independent of the surgical platform. These exclusions may have introduced selection bias, particularly in the economic analysis; therefore, the findings should be interpreted cautiously. RAAD was performed using one of two SRS, depending on the attending surgeon’s discretion and the availability of the surgical setup.

Data collection

Baseline clinical characteristics, perioperative and postoperative outcomes, and surgical and hospitalization costs were compared between the D-RAAD and H-RAAD groups. Clinical data were collected from the NMSH electronic medical records, and all data were extracted by a single investigator using standardized data collection forms. Hospitalization and medical costs per case were analyzed using the Diagnosis Procedure Combination (DPC) data, a unique system in Japan aimed at standardizing and providing efficient, high-quality care at acute-care hospitals nationwide. Medical costs were calculated and converted from Japanese yen to US dollars using the average exchange rate during the study period (1 US dollar = 150 Japanese yen).

Surgical procedure

**Figure 1:**
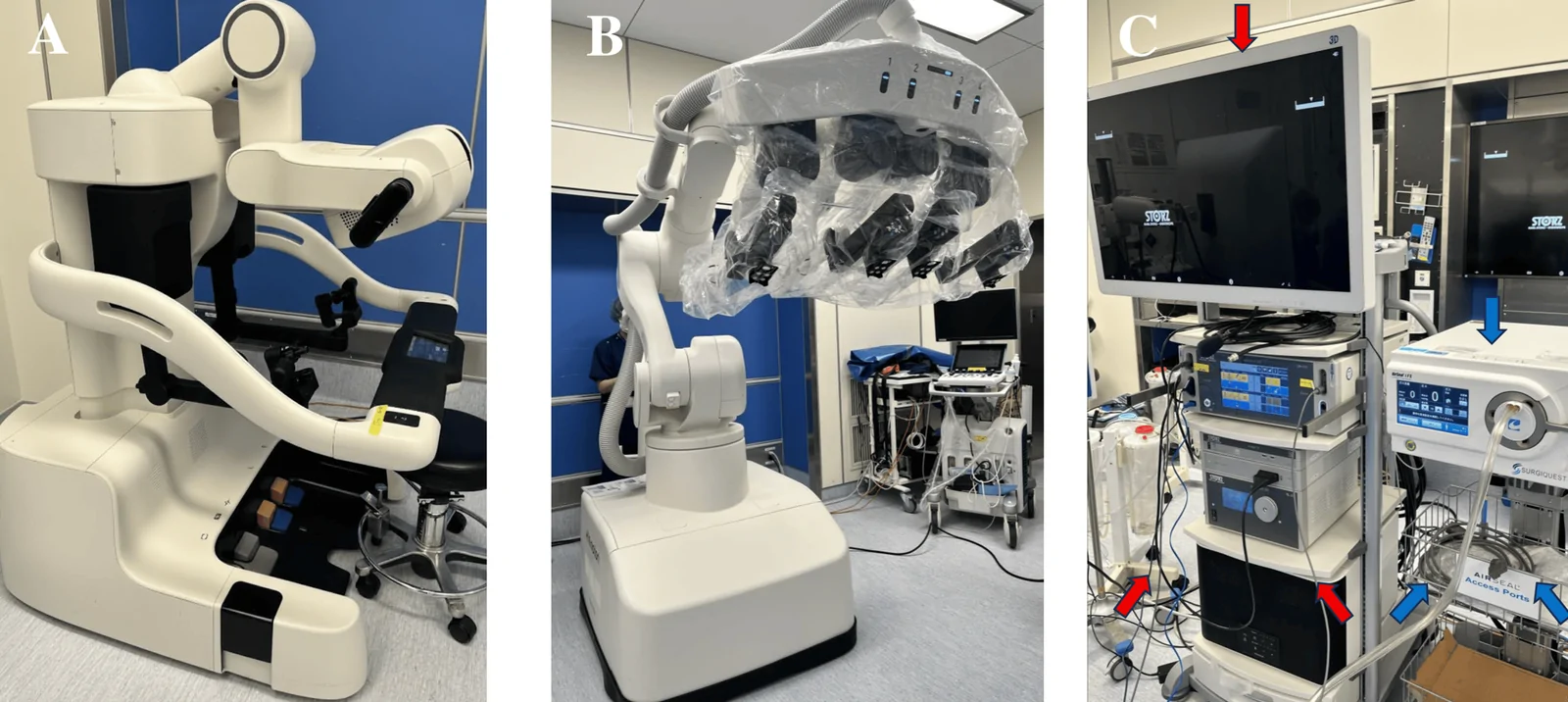
The hinotori™ and AirSeal® intelligent flow system The hinotori™ system showing the (A) surgeon’s cockpit, (B) operation unit, and (C) monitor cart (red arrows) and AirSeal® intelligent flow system (blue arrows).

**Figure 2:**
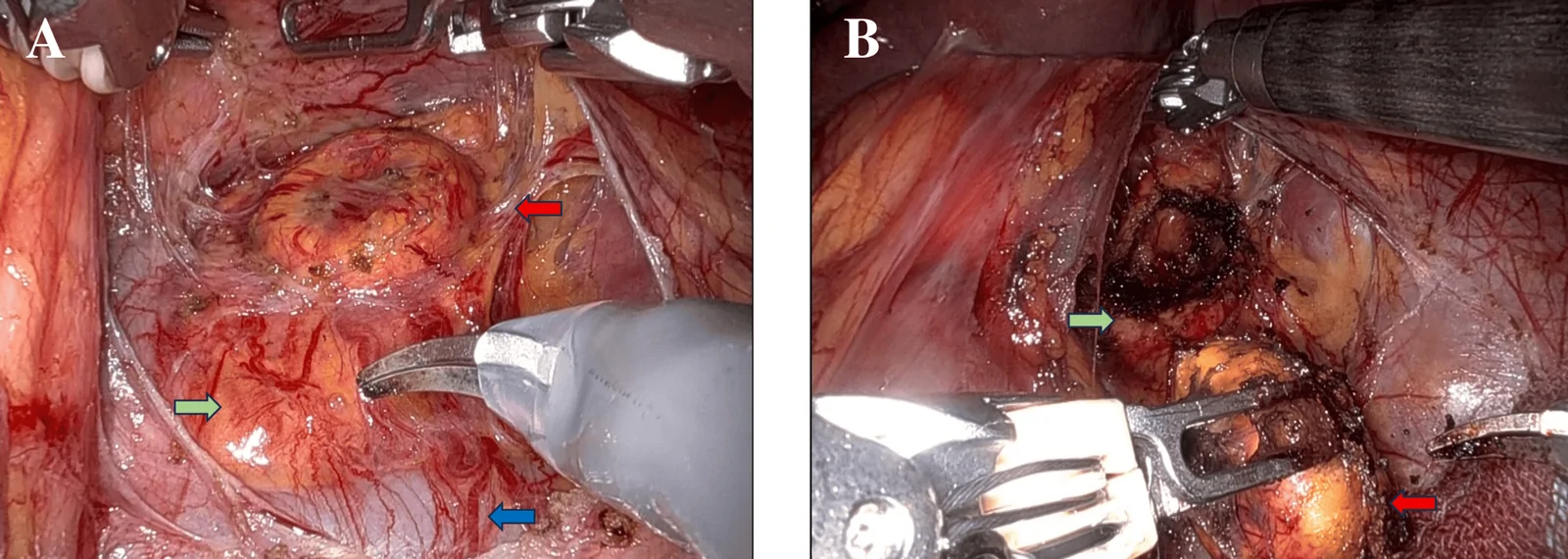
Left partial adrenalectomy (A) Preoperative image, with the red arrow indicating the tumor planned for resection, the green arrow indicating the normal adrenal tissue, and the blue arrow indicating the left renal vein. (B) Postoperative image, with the red arrow indicating the resected tumor and the green arrow indicating the remaining normal adrenal tissue.

RAAD was performed using either the da Vinci Xi™ RAS or the hinotori™ system. Figures 1A and 1B provide an overview of the structural configuration of the hinotori™ surgical system implemented at our center. Five urologists, all certified in both systems, performed RAAD via a transperitoneal approach. In this series, four robotic arms (one camera arm and three instrument arms) and one additional assistant port, incorporating the AirSeal® intelligent flow system (CONMED Japan KK, Tokyo, Japan; Figure 1C), were used for left adrenalectomy. Each surgeon had previously performed approximately 20 robotic prostatectomies before study initiation. For right adrenalectomy, an additional trocar was placed to facilitate liver retraction. In all cases, RAAD was performed using monopolar curved scissors and bipolar fenestrated forceps. For total adrenalectomy, the adrenal vein was transected after applying ML-sized Hem-o-lok® clips (Teleflex Medical Japan, Tokyo, Japan). One patient in each group underwent partial adrenalectomy: one due to patient preference and the other because of young age. A left partial adrenalectomy is illustrated in Figure 2A-2B. In the preoperative image (Figure 2A), the red arrow indicates the tumor planned for resection, the green arrow indicates the normal adrenal tissue, and the blue arrow indicates the left renal vein. In the postoperative image (Figure 2B), the red arrow indicates the resected tumor, whereas the green arrow indicates the remaining normal adrenal tissue.

Outcome measures

Age, body mass index, American Society of Anesthesiologists physical status classification system score [21], history of abdominal surgery, tumor size, and clinical diagnosis were evaluated for each patient group. Perioperative treatment outcomes were assessed according to the duration of general anesthesia, operative time, laparoscopic time, robotic console time, estimated blood loss, duration of antibiotic administration, length of postoperative hospital stay, and time to drainage removal. Complications occurring within 30 postoperative days were assessed using the Clavien-Dindo classification [22]. Because of the small sample size, statistical comparisons were considered exploratory and interpreted cautiously.

Medical expenses based on DPC data were calculated by categorizing them into surgical costs, which included surgical procedure fees and anesthesia management fees, followed by hospitalization costs encompassing meal charges, laboratory and pathology tests, imaging, and nursing staff allocation. Pharmaceutical costs included antidotes, fluids, blood substitutes, hemostatic agents, blood transfusions, general anesthetics, synthetic narcotics, antibiotics, radiocontrast agents, local anesthetics, and skeletal muscle relaxants. Reimbursed materials costs comprised the indwelling bladder catheter, oxygen line, and A‐line monitor. Surgical supplies (non‐reimbursed) included robotic instruments such as scissors, forceps, and graspers, with the cost of robotic instruments being adjusted based on the frequency of SRS use ($560.2 for D‐RAAD and $533.4 for H‐RAAD). Finally, total medical costs were determined as the sum of all these items.

SRS capital and maintenance costs, medical staff expenses, surgical clips, bronchial tubes, suction and irrigation devices, drapes, monitoring sensors for anesthesia and sedation, and warming blankets were excluded because these costs vary substantially among institutions depending on purchasing agreements and utilization volume.

Statistical analysis

The demographic and clinical characteristics of the study population were summarized and analyzed using appropriate statistical methods. Differences between the two groups were compared using Fisher’s exact or Mann-Whitney U tests. Statistical significance was set at p < 0.05. All statistical analyses were performed using R software version 4.3.1 (R Foundation for Statistical Computing, Vienna, Austria). Given the exploratory nature and limited sample size, effect size estimates may be more informative than statistical significance; however, these were not calculated and warrant inclusion in future studies.

Ethics

This study was approved by the NMSH Ethics Committee (approval number: M-2025-401). The requirement for informed consent was waived owing to the retrospective nature of the study and the absence of additional interventions. Participants were allowed to opt out via the NMSH Ethics Committee website.

## Results

Patient characteristics

Baseline clinical characteristics are summarized in Table 1. During the observation period, seven and five patients underwent D-RAAD and H-RAAD, respectively. The H-RAAD group had a median age of 41 (24-70) years and a body mass index of 22.0 (17.1-29.0) kg/m², with one (20.0%) patient having a history of abdominal surgery. Functioning tumors were preoperatively diagnosed in three patients. These characteristics were comparable with those in the D-RAAD group. The median maximum tumor diameter in the H-RAAD group was 20 mm, which was numerically smaller than that in the D-RAAD group.

**Table 1:** Baseline clinical characteristics stratified by type of SRS used ASA-PS: American Society of Anesthesiologists Physical Status [21], BMI: body mass index, FET: Fisher's exact test, U: Mann-Whitney U test, SRS: surgical robotic system

Robotic system (hinotori™/da Vinci Xi™)	Age (years)	Sex	BMI (kg/m^2^)	ASA-PS	Abdominal surgery	Tumor side	Maximum tumor diameter (mm)	Clinical diagnosis
hinotori™ group (n = 5)	26	Male	29	2	Appendectomy	Right	6	Primary aldosteronism
	70	Female	22	2	No	Left	18	Primary aldosteronism
	41	Male	25.1	2	No	Right	44	Non functional
	24	Female	20.8	1	No	Right	23	Cushing's syndrome
	55	Female	17.1	2	No	Right	20	Non functional
Median	41	-	22	-	-	-	20	-
da Vinci Xi™ group (n = 7)	62	Female	23.7	2	No	Left	42	Primary aldosteronism
	58	Female	27.3	2	Caesarean section	Left	42	Non functional
	58	Male	26	2	Duodenal perforation	Left	30	Primary aldosteronism
	53	Female	21.3	3	No	Right	30	Cushing's syndrome
	26	Female	28.8	2	No	Left	37	Non functional
	52	Male	22.7	2	No	Left	45	Non functional
	49	Male	23.9	2	No	Right	34	Primary aldosteronism
Median	53	-	23.9	-	-	-	37	-
Statistical test	U = 12.5	FFT	U = 12.0	FFT	FFT	FFT	U = 6.0	FFT
p-value	0.45	1.00	0.37	0.68	1.00	0.24	0.06	1.00

Clinical data analysis

Table 2 presents the clinical data for all patients. The median anesthesia, total operative, and robotic console times in the H-RAAD group were 264 (253-307) minutes, 189 (156-264) minutes, and 108 (71-180) minutes, respectively. Median operative time was 189 (156-264) vs. 234 (192-252) min (U = 10.0, p = 0.29). Other perioperative parameters, including intraoperative blood loss, length of hospital stay, and duration of antibiotic administration, were similar between the groups. The transperitoneal approach was used for all surgeries, and no patients in either group required conversion to laparoscopic or open surgery. No patient required a blood transfusion or developed complications within 30 days after surgery. No in-hospital deaths or readmissions occurred. One patient in each group underwent partial adrenalectomy.

**Table 2:** Patient safety and perioperative outcome profiles stratified by type of SRS used Values are presented as median (interquartile range) unless otherwise indicated. ADX: adrenalectomy, BT: blood transfusion, CD: Clavien-Dindo [22], EBL: estimated blood loss, FET: Fisher's exact test, Lapa: laparoscopic, OC: open conversion, Ope: operative, U: Mann-Whitney U test, SRS: surgical robotic system

Robotic system (hinotori™/da Vinci Xi™)	Duration of general anesthesia (min)	Ope time (min)	Lapa time (min)	Robotic console time (min)	EBL (mL)	BT	OC	Total or partial ADX	Resected mass weight (g)	Histology	Duration of antibiotic therapy (days)	Duration of drainage (days)	Length of hospitalization (days)	CD grade ≥3	30-day complication	30-day readmission
hinotori™ group (n = 5)	307	242	204	156	108	No	No	Total	13	Adrenocortical adenoma	3	3	7	No	0	0
	254	181	141	82	0	No	No	Partial	1	Adrenocortical adenoma	3	4	8	No	0	0
	326	264	203	180	0	No	No	Total	52	Myelolipoma	3	3	8	No	0	0
	253	156	92	71	0	No	No	Total	14	Adrenocortical adenoma	3	3	9	No	0	0
	264	189	151	108	50	No	No	Total	16	Adrenocortical adenoma	6	3	8	No	0	0
Median	264	189	151	108	0	-	-	-	14	-	3	3	8	-	0	0
da Vinci Xi™ group (n = 7)	302	234	205	154	20	No	No	Total	36	Cystic tumor	3	4	9	No	0	0
	324	247	159	120	0	No	No	Total	44	Adrenocortical hyperplasia with myelolipoma	3	3	8	No	0	0
	319	243	183	135	0	No	No	Total	81	Adrenocortical adenoma	2	4	9	No	0	0
	267	203	162	132	0	No	No	Total	26	Adrenocortical adenoma	3	5	13	No	0	0
	329	252	199	159	0	No	No	Partial	47.2	Ancient neurilemmoma	4	3	7	No	0	0
	271	192	137	117	150	No	No	Total	49.7	Adrenocortical adenoma	3	4	10	No	0	0
	303	230	167	107	0	No	No	Total	31	Adrenocortical adenoma	2	2	8	No	0	0
Median	303	234	167	132	0	-	-	-	44	-	3	4	9	-	0	0
Statistical test	U = 10.0	U = 10.0	U = 14.0	U = 14.0	U = 15.0	-		FFT	U = 6.0	-	U = 24.0	U = 12.5	U = 17.5	-		-
p-value	0.22	0.29	0.57	0.57	0.70	1.00	1.00	1.00	0.06	-	0.29	0.38	1.00	1.00	1.00	1.00

Costs analysis

The medical costs for each group were evaluated according to the type of SRS used. The median costs per case for the surgical, hospitalization, pharmaceutical, reimbursed material, and non-reimbursed surgical supply categories for H-RAAD and D-RAAD are shown in Table 3. Surgical costs tended to be lower in the H-RAAD group, whereas hospitalization costs tended to be lower in the D-RAAD group. Median total medical costs per case were $6,333.1 for D‐RAAD and $6,398.4 for H‐RAAD; these values were similar between the groups (U = 23.0, p = 0.34).

**Table 3:** Cost analysis stratified by type of SRS used U: Mann-Whitney U test, SRS: surgical robotic system

Robotic system (hinotori™/da Vinci Xi™)	Surgical costs ($)	Hospitalization costs ($)	Pharmaceutical costs ($)	Reimbursed materials costs ($)	Surgical supplies non-reimbursed materials ($)	Total costs ($)
hinotori™ group (n = 5)	2651.3	2528.1	225.9	82.5	711.1	6198.9
	3405.0	2805.5	228.2	82.3	533.4	7054.4
	2625.3	2751.3	208.9	42.8	466.1	6094.5
	3330.0	2905.2	222.8	43.3	533.4	7034.7
	2480.7	3115.4	224.8	44.1	533.4	6398.4
Median	2651.3	2805.5	224.8	44.1	533.4	6398.4
da Vinci Xi™ group (n = 7)	3448.0	1864.1	212.1	81.8	748.7	6354.7
	3489.0	1735.6	274.0	81.4	560.2	6140.3
	3394.0	2242.4	195.7	45.4	492.9	6370.4
	3706.3	1647.7	195.7	71.7	492.9	6114.3
	3618.0	1813.2	132.9	129.7	681.3	6375.1
	3605.0	1770.2	148.4	128.1	681.3	6333.1
	3437.0	1893.7	170.1	127.9	492.9	6121.5
Median	3489.0	1813.2	195.7	81.8	560.2	6333.1
Statistical test	U = 1.0	U = 35.0	U = 28.0	U = 10.0	U = 15.0	U = 23.0
p-value	0.005	0.003	0.07	0.15	0.74	0.34

## Discussion

We evaluated the clinical outcomes and associated medical costs after the introduction of H-RAAD at an academic center in Japan. Our results suggest that H-RAAD can be performed without major perioperative safety concerns in selected patients, with perioperative outcomes and reimbursed medical costs comparable with those of D-RAAD in this small exploratory cohort.

RAAD has increasingly been adopted as a minimally invasive surgical approach for adrenal tumors, offering potential advantages over conventional laparoscopic techniques. A comprehensive pooling of data by Gan and colleagues, which aggregated findings from 26 comparative studies involving nearly 3,000 cases, underscored the clinical benefits of the robotic approach over conventional laparoscopy [23]. Their meta-analysis revealed that patients undergoing robot-assisted surgery experienced significantly lower intraoperative blood loss, expedited hospital discharge, and a reduced risk of conversion to open surgery. To date, the available evidence on RAAD has been largely derived from studies using the da Vinci Xi™ system; however, early clinical experiences with RAAD using newly developed SRS have been increasingly reported. Preliminary single-center analyses comparing D-RAAD with Hugo™-assisted RAAD showed that clinical outcomes were comparable [24]. Additionally, a single-center case series (n = 12) involving Senhance™-assisted RAAD reported technical success [25].

Furthermore, several reports have described RAAD using the KangDuo™ and Revo-i™ systems, demonstrating procedure feasibility along with acceptable safety and efficacy profiles [15,16]. Using the hinotori™ system, Motoyama et al. reported an early clinical experience comparing six H-RAAD cases with five D-RAAD cases [26]. All H-RAAD surgeries were completed safely without conversion, with acceptable operative times, minimal blood loss, and no major complications. In the present study, we conducted a clinical investigation of RAAD performed at a single institution in Japan and observed acceptable perioperative outcomes. The study cohort included a diverse range of adrenal pathologies, such as aldosterone-producing adenomas, Cushing’s syndrome, and large tumors. In this series, all procedures were completed without conversion or major complications across these pathologies. As the introduction of additional novel SRS is anticipated, these findings may contribute to the accumulation of early clinical evidence regarding H-RAAD within the Japanese healthcare setting.

The D-RAS system has played a central role in the development of SRS since receiving approval from the United States Food and Drug Administration in 2000. The development of novel SRS has accelerated, fostering technological innovation while promoting a competitive environment aimed at reducing healthcare costs. To date, multiple cost analyses of robot-assisted radical prostatectomy (RARP) using novel SRS have been reported [27]. Studies evaluating systems such as the Senhance™, KangDuo™, Revo-i™, and Mantra™ have demonstrated cost-effectiveness comparable to that of the conventional da Vinci™ system. At NMSH, we have also previously demonstrated comparable economic outcomes of RARP between the hinotori™ and da Vinci Xi™ systems [28]. RAAD is reported to be approximately 2-2.5 times more expensive than laparoscopic adrenalectomy, largely due to longer operative times and robot-related expenses [29]. However, cost analyses of RAAD are limited, and there are no published cost evaluations of individual SRS, primarily because RAAD is performed far less frequently than RARP. To our knowledge, this is the first study to compare the economic impact of H-RAAD and D-RAAD in Japan. Given the relative rarity of RAAD, referral to high-volume centers may improve procedural efficiency and optimize healthcare resource utilization.

Although several cost categories differed numerically between the groups, total reimbursed medical costs were comparable. These differences may reflect variations in perioperative management during the early implementation phase of the hinotori™ system. They should be interpreted cautiously given the limited sample size and potential influence of the institutional learning curve. Because these costs were derived from the Japanese DPC reimbursement system, extrapolation to healthcare systems with different reimbursement structures should be undertaken cautiously.

This study had several limitations. First, it was conducted at a single institution with a limited number of patients undergoing D-RAAD and H-RAAD. Consequently, this study should be interpreted as an exploratory analysis rather than a definitive comparative evaluation. In high-volume centers, reduced operative time and instrument use may be achieved with experienced surgical teams, thereby mitigating overall costs [30]. Second, the H-RAAD group had a smaller median tumor diameter, which may have influenced operative complexity and perioperative outcomes. Third, the economic analysis of RAAD did not account for SRS capital and maintenance costs. Fourth, the number of assistant trocars differed according to tumor laterality: two were placed for right adrenal tumors, whereas one was placed for left adrenal tumors. Finally, RAAD was performed by five surgeons with varying levels of RAS experience, which may have affected operative time, perioperative management, and associated medical costs. Because this study was retrospective and exploratory, the absence of statistically significant differences should not be interpreted as evidence of equivalence or non-inferiority between the two robotic systems. This is one of the few investigations to evaluate both the clinical outcomes and economic implications of introducing H-RAAD.

## Conclusions

This exploratory study demonstrates that H-RAAD is clinically feasible and safe, yielding perioperative outcomes comparable to those of the established da Vinci Xi™ system. Furthermore, total reimbursed medical costs under the Japanese DPC system were similar between the two platforms. These findings suggest that the introduction of domestically developed SRS may represent a feasible alternative for minimally invasive adrenal surgery. However, given the small sample size and the exclusion of capital and maintenance costs, these results remain preliminary. Larger, multicenter cohorts with comprehensive health economic evaluations are required to definitively establish the clinical and economic equivalence of these platforms.
